# Spironolactone hyaluronic acid enriched cerosomes (HAECs) for topical management of hirsutism: *in silico* studies, statistical optimization, *ex vivo,* and *in vivo* studies 

**DOI:** 10.1080/10717544.2021.1989089

**Published:** 2021-11-02

**Authors:** Rofida Albash, Abdurrahman M. Fahmy, Mohammed I.A. Hamed, Khaled M. Darwish, Rania Moataz El-Dahmy

**Affiliations:** a Department of Pharmaceutics, College of Pharmaceutical Sciences and Drug Manufacturing, Misr University for Science and Technology, Giza, Egypt; b Department of Pharmaceutics and Industrial Pharmacy, Faculty of Pharmacy, Cairo University, Cairo, Egypt; c Department of Organic and Medicinal Chemistry, Faculty of Pharmacy, Fayoum University, Fayoum, Egypt; d Department of Medicinal Chemistry, Faculty of Pharmacy, Suez Canal University, Ismailia, Egypt; e Department of Pharmaceutics and Industrial Pharmacy, Faculty of Pharmacy, October 6 University, Cairo, Egypt

**Keywords:** *In silico* study, spironolactone, hirsutism, dermatokinetic, local accumulation efficiency index

## Abstract

Spironolactone (SP) is a potassium sparing diuretic with antiandrogenic properties. This study aimed at formulating SP into hyaluronic acid enriched cerosomes (HAECs) for topical management of hirsutism. HAECs were prepared by ethanol injection method, according to D-optimal design, after a proper *in silico* study. HAECs were evaluated by measuring their entrapment efficiency (EE%), particle size (PS), and polydispersity index (PDI). Optimal hyaluronic acid enriched cerosomes (OHAECs) were subjected to further *in vitro* and *ex-vivo* and *in-vivo* studies. The *in silico* study concluded better interactions between SP and phosphatidyl choline in presence of hyaluronic acid (HA) and high stability of their binding in water. The prepared HAECs had acceptable EE%, PS, and PDI values. The statistical optimization process suggested OHAEC containing 10.5 mg ceramide III and 15 mg HA, utilizing Kolliphor^®^ RH40. OHAEC had EE% and PS of 89.3 ± 0.3% and 261.8 ± 7.0 nm, respectively. OHAEC was stable for up to 3 months. It also showed a mixed tubular and vesicular appearance under transmission electron microscope. The *ex vivo* and *in vivo* studies concluded better skin deposition and accumulation of SP from OHAEC. The histopathological study demonstrated the safety of OHAEC for topical application. Therefore, OHAEC could be considered as effective system for topical application of SP to manage hirsutism, with prolonged action, coupled with minimized side effects.

## 1. Introduction

Hirsutism is a common disorder in females, usually linked to polycystic ovarian syndrome and other causes. It is characterized by the abnormal presence of hair in unwanted areas (Van Zuuren & Fedorowicz, 2016; Azarchi et al., 2019). Spironolactone (SP) is a potassium-sparing diuretic used for edema (Rathnayake & Sinclair, 2010). It has been found to have antiandrogen effect via reducing androgens production and competitively blocking their receptors. Dermatologists prescribe SP (200 mg daily, orally) for alopecia and hirsutism, but such high doses can cause systemic side effects (Burke & Cunliffe, 1985; Rathnayake & Sinclair, 2010). This encouraged different researchers to develop SP topical formulations to increase its local activity at hair follicles, together with the aim of reducing its systemic side effects. In 2012, Afzali et al. (2012) proved the efficacy of a 5% SP-gel in reducing total acne lesions (TLC) compared to a plain gel, but with no significant difference for acne severity index (ASI). In 2014, Shamma & Aburahma (2014) developed SP-loaded nanostructured lipid carriers (NLCs). All the formulae were in the nano range with entrapments of >74%. They demonstrated an initial burst release of SP, followed by sustained release. The confocal laser scanning microscopy confirmed the ability of the nanocarriers to localize SP within hair follicles. In 2021, Amer et al. (2021) developed topical gel containing NLCs of both SP and progesterone. All formulations were in the nano range with entrapments of >75% and highly negative zeta potential (ZP). The application of the gel for 21 days reduced the induced hirsutism in female rats. In the same year, Ilic et al. (2021) utilized emulsions with alkyl polyglucosides as a vehicle for SP topical delivery. The gels showed acceptable irritation profiles with good ability to hydrate the skin, together with acceptable stability. Cerosomes gained interest recently as topical drug delivery platforms. They have been employed by Abdelgawad et al. (2017) and Albash et al. (2021) for the successful topical delivery of tazarotene and fenticonazole nitrate, respectively. In the earlier study, the researchers included ceramide VI with phospholipid with or without Tween 80 or sodium deoxycholate. The obtained cerosomes had high entrapment efficiencies (58–99%), together with suitable viscosities and sustained release of tazarotene. In the latter study, the researchers included ceramide IIIB with phospholipid and PEGylated surfactants (Brijs). The optimal formula showed desirable *in vitro* characteristics (83% entrapment efficiency, 551.6 nm particle size, and 20.9 mV zeta potential) and was superior to fenticonazole nitrate suspension in localizing the drug within rat skin layers. The aim of this study was to formulate and evaluate hyaluronic acid enriched cerosomes (HAECs) that are nanostructures composed of ceramides, phospholipid, hyaluronic acid and edge activator (Kolliphor RH40 or Kolliphor EL). Each of these components had one or more function. In more details, ceramides may provide protective activity and skin hydration. Addition of edge activators in the formulations enhances the drug deposition and aids in the production of highly stable unaggregated vesicles of the double lipidic, phosphatidylcholine–ceramide mixture. Both components are expected to augment the topical effect of SP. Hyaluronic acid was used to enhance the nanovesicles bioadhesion. *In silico* studies are computer aided studies used to predict the interaction between molecules and their behavior in different media, respectively. So, this study was targeted to formulate SP-HAECs, that might have acceptable dermal deposition profile for topical management of hirsutism.

## 2. Materials and methods

### 2.1. Materials

SP was supplied by SEDICO, Pharmaceuticals Co., (Cairo, Egypt). Hyaluronic acid (HA), kolliphors RH 40 and EL were purchased from Acros Organics, Belgium. L-α phosphatidyl choline (PC) was purchased from Sigma Aldrich, USA. Ceramide III was provided by Evonik Co. (Germany). Chloroform, ethanol, and methanol were purchased from El-Nasr Pharmaceutical Company, Cairo, Egypt.

### 2.2. Methods

#### 2.2.1. Molecular docking and molecular dynamics simulation (MDS)

An *in silico* study used to predict the binding of SP and PC in presence of HA using a molecular docking study. Additionally, the thermodynamic stability of SP-PC-HA (SPH) system was investigated throughout an all-atom MDS to choose suitable solvent(s) for preparation of HAECs and predict their stability in water. The molecular docking workflow involved 3 D construction of SP, PC, and HA using the builder module within the Molecular Operating Environment (MOE) software (Chemical Computing Group Incorporation, Montreal, Canada). Partial charges were assigned, and minimization was proceeded using the Amber10: EHT forcefield to an energy gradient of 0.1 RMS Kcal mol^−1^ A^−2^. SP and HA were docked on the PC. The docked ligand conformations were developed through bond rotation, lodged within in the active site guided by triangular-matching approach, then conformations were ranked via the London_dG scores. The top ten docked poses (*n* = 10) were retained for refinement then an energy minimization stage, within the target pocket, before they were rescored by Generalized-Born solvation-VI/Weighted-Surface Area_dG (GBVI/WSA) force field scorings. The MOE incorporated scoring system relies on Coulomb’s electrostatics using protein-ligand van der Waals score, current-loaded charges, exposure-weighted surface area, and solvation electrostatics (Vilar et al., 2008). The predicted binding pose for the SPH model was selected based on the highest docking energy scores (the more negative Kcal mol^−1^ values) as well as favored polar and hydrophobic binding interaction. The cutoff values for hydrogen bond (donor-H…acceptor; DH-A) distance and angle were assigned at 3.0 Å and 20°, respectively (de Souza et al., 2019; Albuquerque et al., 2020), while as the hydrophobic interactions (≤5 Å) were determined via the MOE ligand interactions tool, in addition to manual measurements done via the PyMol bond distance measurement tools. The top-docked SPH pose was then proceeded through an explicit MDS using the simulation module incorporated within the MOE software. The SPH model was initially minimized to a low gradient of 1 × 10^−5^ RMS Kcal mol^−1^ A^−2^. Subsequently, the minimized systems were separately fully solvated within a 3 D cubic box (44.7 Å in all directions *x*, *y*, *z*) containing water, chloroform, or both ethanol and chloroform using the explicit Amber10:EHT forcefield. The periodic boundary conditions were applied in all dimensions maintaining a 10 Å non-bounded cutoff distances. The composition of each established system is presented within Table 1. Following system setup, system minimization and equilibration was proceeded through applying full-energy minimization and subsequent two-step equilibration stages under initial constant number of particles, volume, and temperature (NVT) and subsequent constant number of particles, pressure, and temperature (NPT) ensembles using the Nosé–Hoover–Andersen equations of motion (Allen & Schmid, 2006). A single-step minimization was done for 500 ps, followed by 500 ps equilibration under each NVT and NPT ensembles at 310 K and 310 K/101 kPa, respectively. Finally, each minimized/equilibrated system was run for 1 ns. The molecular dynamics trajectories were analyzed by the MOE database calculator for estimating the interaction potential energy between PC and both SP-HA. On the other hand, snapshots at 0.2, 0.4, 0.6, 0.8, and 1 ns were represented by the PyMol ver. 2.0.6 software (Schrödinger™, New York, USA) for examining the time evolution of the SPH conformational changes.

**Table 1.** Atomic composition of the investigated SP-PC-HA simulated systems.

Solvation State	composition (Number of molecules)						
	SP	PC	HA	Chloroform	Ethanol	Water	Total
100% Water	1	1	1	–	–	4053	4056
100% Chloroform	1	1	1	704	–	–	707
Chloroform + ethanol	1	1	1	352	635	–	990

Abbreviation: SP, Spironolactone; PC, phospholipid, and HA, hyaluronic acid.

#### 2.2.2. Experimental design

SP-HAECs were prepared according to D-optimal design using Design-Expert^®^ software (Stat-Ease, Inc., Minneapolis, MN) (Table 2) to study the effect of variables on the vesicles’ properties to reduce the number of experimental runs needed for conducting the study (Nemr et al., 2021; Radwan et al., 2020).

**Table 2.** D-optimal design used for optimization of SP-loaded HAECs.

Factors (independent variables)	Factor type	Levels	
		(−1)	(+1)
*X*_1_: Ceramide amount (mg)	Numeric	5	15
*X*_2_: HA (mg)	Numeric	5	15
*X*_3_: EA type	Categoric	Kolliphor RH40	Kolliphor EL

Responses (dependent variables)	Desirability constraints
*Y*_1_: EE%	Maximize
*Y*_2_: PS (nm)	Minimize
*Y*_3_: PDI	Minimize

Abbreviation: HA: hyaluronic acid; EA: edge activator; EE%: entrapment efficiency percent; SP: Spironolactone; PS: particle size; PDI: polydispersity index and HAECs; hyaluronic acid enriched cerosomes.

#### 2.2.3. Fabrication of SP-HAECs using ethanol injection method

SP-HAECs were prepared using ethanol injection method (Kakkar and Kaur, 2011). SP, PC, ceramide, and the EAs (Kolliphor RH40 or EL) were dissolved in 4 mL of a 1:1 ethanol/chloroform mixture. The organic mixture was introduced into a hot (60 °C) aqueous phase (10 mL) in which HA had been pre-dissolved. The formed mixture was stirred on a magnetic stirrer (Model MSH-20D, GmbH, Germany) for 30 min at 800 rpm then formulae were kept at refrigerator.

#### 2.2.4. Determination of percentage entrapment efficiency (EE%)

About 1 mL of the formula was subjected to ultracentrifugation at 22000 rpm and 4 °C for 1 h using ultracentrifuge (model 3-30KS; Sigma Zentrifugen, Germany), followed by lysis using methanol. The UV absorbance was measured using spectrophotometer (model UV-1601 PC; Shimadzu, Kyoto, Japan), at the predetermined *λ*_max_ (256 nm). The EE % was calculated by the direct method (Al-Mahallawi et al., 2017).

#### 2.2.5. Determination of particle size (PS), polydispersity index (PDI), and zeta potential (ZP)

Zetasizer (Nano ZS, Malvern Panalytical Ltd, UK) was used to measure the PS and PDI. The formulae were diluted before measurement (Scognamiglio et al., 2013; Abd-Elsalam et al., 2018). ZP of the optimal formula was determined using the same equipment by tracking the mobile particles in electric field (Abd-Elsalam et al., 2018).

#### 2.2.6. Optimization of SP-HAECs based on the desirability criterion

An optimal formula was to be suggested based on the desirability criterion, and according to the constraints listed in Table 2, using Design-Expert- 7^®^ software. This formula was prepared and characterized. The predicted and observed responses were recorded, then correlated through calculation of the bias percent by using the equation (Abdel-Hafez et al., 2018):

(1) $$\text{Bias percent }=\;\frac{|\;\mathrm{Predicted}\;\mathrm{value}-\mathrm{observed}\;\mathrm{value}\;|\;}{\mathrm{observed}\;\mathrm{value}}\times \;100$$

#### 2.2.7. Further *in vitro* characterization of OHAECs

##### 2.2.7.1. Effect of short-term storage

OHAEC formula was stored for 3 months in refrigerator. The formula was observed for changes in visual appearance. EE%, PS, ZP, and PDI were compared using Student *t*-test using Statistical Package for the Social Sciences (SPSS^®^) V.17 at *p* < 0.05 before and after storage.

##### 2.2.7.2. Morphology of OHAECs

Transmission electron microscope (TEM) was utilized for the examination of the morphology of optimal hyaluronic acid enriched cerosomes (OHAECs). The formula was diluted then deposited in a carbon-coated copper grid and stained by 2% w/v phosphotungestic acid (Fahmy et al., 2018).

#### 2.2.8. *Ex vivo* permeation and deposition studies

Newly born rats were sacrificed and then the dorsal skin was removed (Kumar et al., 2007). The skin was frozen at −20 °C (Hashem et al., 2011). The permeation of SP, through skin, from OHAECs, compared to SP suspension was conducted. The skin was mounted on a plastic dialysis tube with permeation area of 3.14 cm^2^. About 1 mL from OHAECs equivalent to 1 mg SP was introduced into the donor compartment while the permeation medium was 50 mL of phosphate buffer (pH= 5.5) at 37 ± 0.5 °C stirred at 100 rpm. Samples of 1 mL were withdrawn at 1, 2, 4, 6, 8 and 24 h and then analyzed using HPLC. The cumulative amounts permeated per unit area were calculated (Kelidari et al., 2017; Fahmy et al., 2018). Afterward the maximum flux values at 24 h (*J*_max_) were obtained (Elgorashi et al., 2008). The skin was separated and washed, vortexed with 5 mL methanol. Skin was subjected to one cycle of sonication for 90 min then subjected to centrifugation at 10,000 rpm for 15 min, then SP concentration was determined by HPLC to obtain the amount deposited after 24 h (Dep_24_) (Radwan et al., 2017). Finally, the local accumulation efficiency index (LAEI) for SP were obtained as the ratio of SP accumulated into the skin to that delivered through the skin (El-Hadidy et al., 2012).

#### 2.2.9. *In vivo* studies

##### 2.2.9.1. Handling of animals

Male Wistar rats (150–200 gm), with an average age of 7 weeks were used. The study protocol was approved by Research Ethics Committee (REC) for experimental and clinical studies at Faculty of Pharmacy, Cairo University. For the administration of SP suspension and OHAECs through *in-vivo* studies, bottle caps were utilized as drug pools with an area of 4.91 cm^2^. The bottle caps were fixed to dorsal rat skin which was shaved previously (Abdelbary and AbouGhaly, 2015).

##### 2.2.9.2. Histopathological studies

The rats were divided into three groups with two rats each. Group I acted as control. Rats in group II were subjected to SP suspension, while rats in group III had OHAECs applied to their skin, three times daily for 1 week. The rats were then sacrificed, and the skin was excised. Skin samples were fixed in 10% formaline, embedded in paraffin. Sections from the paraffin blocks were cut using a microtome (Leica Microsystems SM2400, Cambridge, England). The obtained sections were stained, then examined by light microscope (Abdelbary & AbouGhaly, 2015).

##### 2.2.9.3. Dermatokinetic study

Animals were divided into 2 groups of 18 animals each. One group received SP suspension and the other obtained the OHAECs topically. Half mL of each test formula (equivalent 0.5 mg SP) was applied to rat skin. After treatment, three animals from each group were sacrificed at 1, 2, 4, 6, 8, and 10 h. The excised skin was cut into pieces and sonicated in 5 mL methanol for 30 min. The extract was then filtered, and the concentration of SP was determined by HPLC. AUC_0–10_ was calculated for each test formula using Kinetica^®^ 5 software (Thermo Fisher Scientific Inc., Waltham, MA, USA). AUC_0–10_ for both treatments was compared using student t-test by SPSS^®^ software 22.0.

## 3. Results and discussion

### 3.1. Molecular docking and MDS

**Figure 1.**
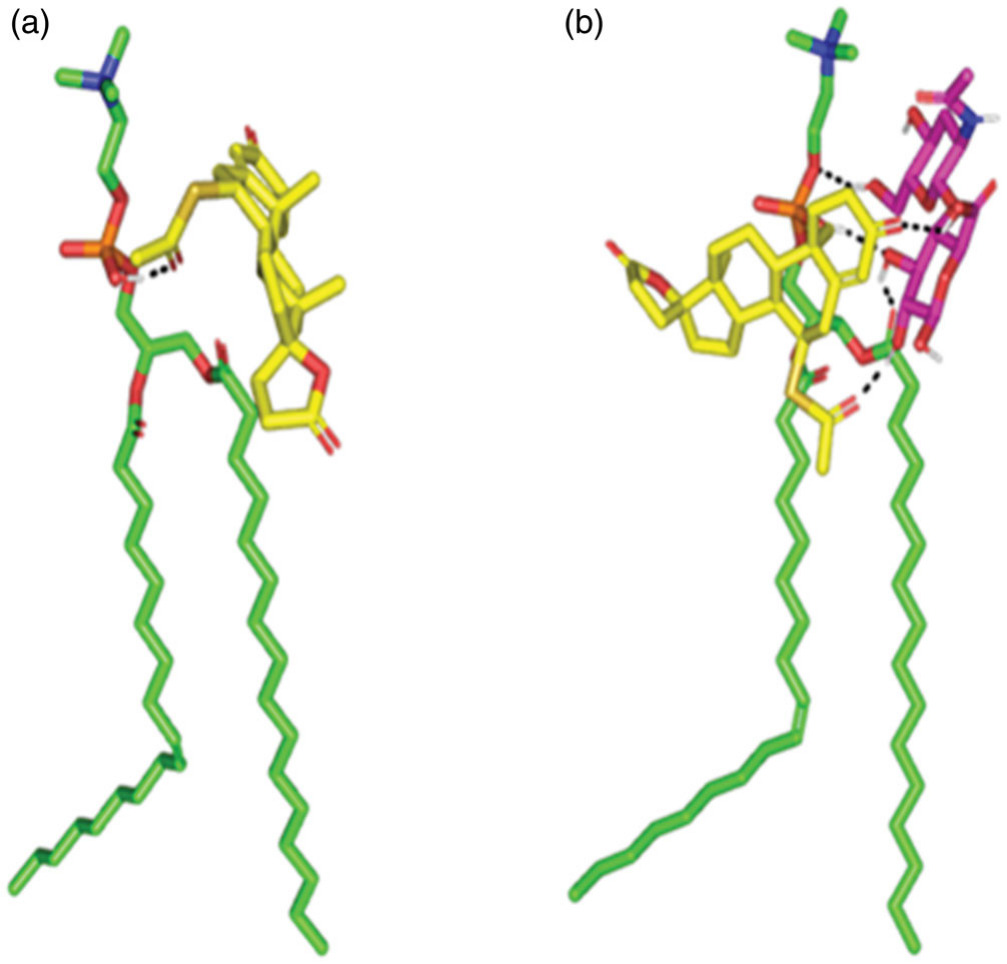
Binding poses of the spironolactone phosphatidyl choline docked complex. (a) In absence of the hyaluronic acid monomer. (b) In combination with the hyaluronic acid monomer (magenta sticks) serving as an anionic formulation additive. Predicted binding interactions are illustrated between the drug molecule; spironolactone (yellow sticks) and of the hyaluronic acid monomer; phosphatidyl choline (green sticks). More favored complex stability was assigned for the spironolactone–phosphatidyl choline in combination with hyaluronic acid. Polar interactions (hydrogen bonding), discussed within the text, are depicted as black dashed lines.

**Figure 2.**
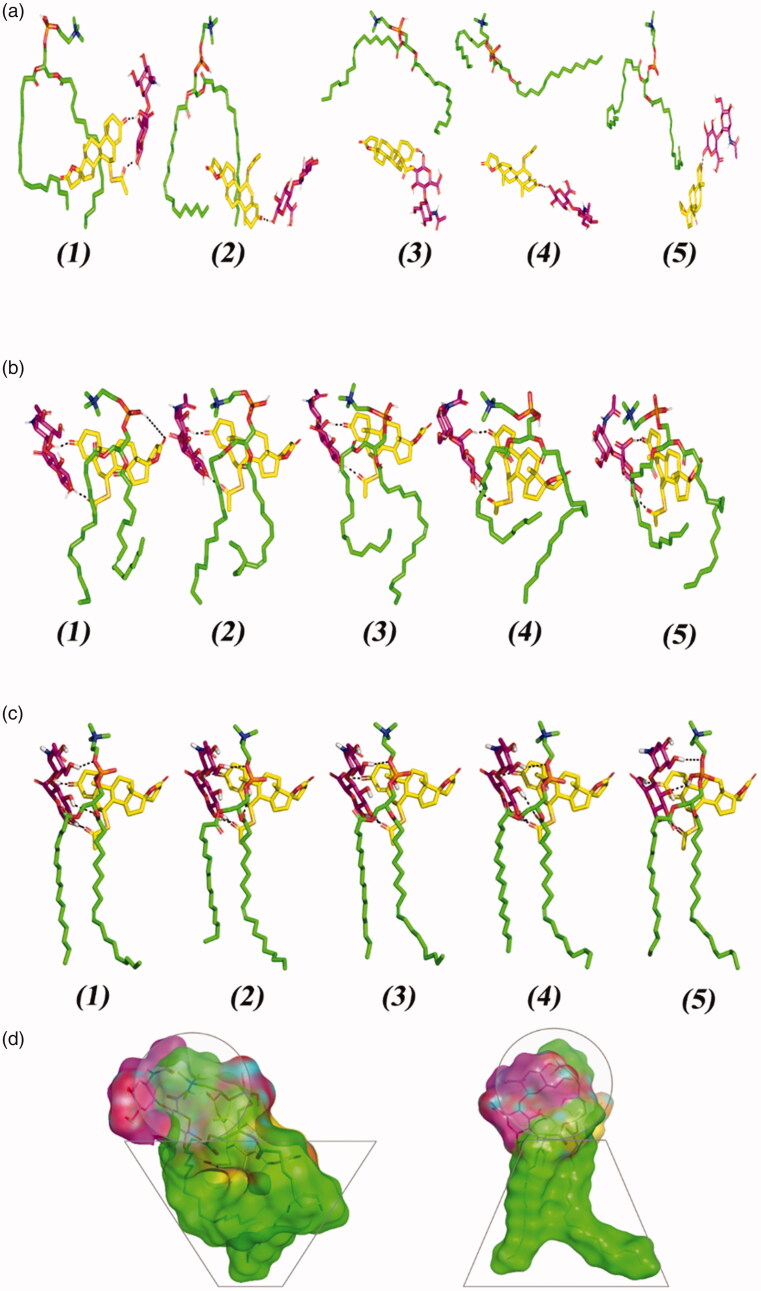
Conformation alterations time evolution of SPH ternary complex throughout the explicit molecular dynamics simulation at different solvation system. The thermodynamic movements and stability of formulation components; spironolactone (yellow sticks), hyaluronic acid monomer (magenta sticks), and phosphatidyl choline (green sticks) were monitored over MD simulation trajectories within (a) 100% chloroform; (b) combined chloroform/alcohol; (c) 100% water solvated systems at captured at different snapshots (1) 0.2 ns; (2) 0.4 ns; (3) 0.6 ns; (4) 0.8 ns; and (5) 1 ns. Polar interactions (hydrogen bonding) are depicted as black dashed lines. (d) Molecular surface 3 D representation of the cone micellar configuration of optimized SPH in chloroform: alcohol (1:1) combination (left panel), as well as the inverted cone micellar configuration of 100% water (right panel). Molecular surface representations were illustrated in colors being previously assigned for the optimized SPH components; yellow, magenta, and green are for spironolactone, hyaluronic acid monomer, and phosphatidyl choline, respectively.

The molecular docking revealed the interaction between SP and PC. In absence of HA, the SP favor interacted near the phosphate head of the PC via its acetylthio substitution while lying the part of SP structure toward the hydrophobic PC extended acyl chains (Figure 1(a)). Despite that SP possesses several hydrogen bond acceptors (*n* = 5), only single polar interaction (hydrogen bonding) was depicted toward the PC since the latter only furnish a single hydrogen bond donor at its phosphate functionality (–OPO(O)OH). The latter predicted docking pose came in good agreement with reported literatures showing preferential binding of several drugs: including metformin and rosuvastatin, at the phosphate head of the PC molecule (Abd-Elsalam et al., 2018; Farag et al., 2021). However, the skewness of the SP structure toward the hydrophobic PC extended acyl chains was reasoned since SP incorporates a highly hydrophobic cage-like core skeleton resembled in its steroidal nucleus. Combining the hydrophobic interactions and the single polar contact between SP and PC could not build up a significant binding interaction. In these regards, poor docking score was depicted for the SP-PC complex (−3.307 kcal/mol) inferring unfavored binding potentiality. On the other hand, docking of SP on the PC in presence of HA revealed differential binding interactions. Owing to the high number of hydrogen bond donners and acceptors, the anionic HA managed to form several polar contacts with both the SP and PC (Figure 1(b)). At a singular interface of the PC molecule, two strong hydrogen bonds were mediated by the C_3_ hydroxyl group of the d-glucuronic acid moiety toward the free OH of the PC phosphate group (hydrogen bond distance/angle = 2.2 Å/141.3°) and the carbonyl group of the hydrophobic acyl tail (2.1 Å/135.8°). An additional polar interaction was furnished between the HA C_6_-OH at the *N*-acetyl-d-glucosamine scaffold and oxygen atom of the phosphate group (2.3 Å/152.0°). The d-glucopyranosyl moiety of the HA further mediated strong polar contacts with the acetylthio group and C_3_ carbonyl functionality of the SP skeleton (2.0 Å/163.6° and 2.3 Å/121.6°, respectively). On the other hand, proximity hydrophobic contacts were illustrated by SP spiro rings and acetylthio substitution at the PC non-polar tails (<5 Å). Based on the extended polar network and strong hydrophobic interactions among the formulation main three components, higher docking score was obtained with a combined value of −6.566 kcal/mol suggesting higher drug binding stability on the PC. The above obtained docking findings could explain the importance of the introduction of HA. In addition, HA would serve as a carrier mediating the loading of SP on the PC molecule for optimized SP EE%. The dispersion behavior and thermodynamic stability of the SPH tertiary complex within water, chloroform, ethanol, and 1:1 stoichiometric combination of chloroform: ethanol was investigated through an explicit MDS. Moreover, MDS within 100% chloroform showed significant instability of the SPH complex (Figure 2(a)). Following initial simulation time (at 0.2 ns), the HA lost hydrogen bonding with the PC phosphate head while maintaining strong polar contacts of two hydrogen bonds with the SP carbonyl oxygens (1.6 ± 0.32 Å/148.3 ± 0.11° and 2.1 ± 0.33 Å/124.8 ± 0.85°). The latter dynamic behavior was correlated with a significant drop of the interaction potential energy (both electrostatic and van der Waals potentials) between SP, HA, and PC across the MDS run (−75.182 Kcal/mol at 0 ns down to −62.314 Kcal/mol at 1 ns; average sum-over-states binding energy = −69.187 ± 5.65 Kcal/mol). Thus, the stability of the SPH complex was compromised in 100% chloroform. Since addition of ethanol throughout the formulation development stage was essential to bring chloroform and water into contact, investigating the SPH thermodynamic stability and conformational alteration was proceeded through ethanol: chloroform solvated system. Notably, both the SP and HA were stable at the PC head across the MDS run (Figure 2(b)), with average sum-over-states binding energy (−128.73 ± 13.87 Kcal/mol) which was nearly two-fold higher than that of 100% chloroform, thus favoring the use of a mixed solvent during the preparation process. To estimate the stability of binding in the final formula (100% water), the SPH complex was significantly stable with average sum-over-states binding energy (−212.14 ± 12.53 Kcal/mol). Several polar contacts were maintained along the whole MDS between the SP and HA (Figure 2(c)). Double hydrogen bonds were depicted between the C_1_–OH and C_2_–OH of the acid sugar in one hand and the carbonyl group of the drug acetylthio substitution at the other hand (average 1.9 ± 0.31 Å/139.5 ± 0.22° and 1.6 ± 0.12 Å/155.1 ± 0.65°). Moreover, strong hydrogen bond was illustrated between the carboxylic group of HA nucleus and the 3-oxo group of SP (average 1.5 ± 0.08 Å/153.9 ± 0.73°). These polar contacts maintained tight closeness between the formulation sugar additive and SP nucleus. On the other hand, the hydroxyl groups at C_3_ of the glucuronic acid and C_1_ of the *N*-acetyl d-glucosamine portion of the HA-mediated alternating polar contacts with the phosphate group (average 2.5 ± 0.05 Å/144.9 ± 0.19° and 2.8 ± 0.27 Å/125.6 ± 0.11°, respectively) as well as the acetyl moiety of one of the hydrophobic tails (average 2.6 ± 0.07 Å/153.3 ± 0.27°) across the MDS. Such polar contacts allowed pulling both PC hydrophobic acyl tails away from each other furnishing an opened compass conformation which would have increased the volume of the hydrophobic chain. On the contrarily, small surface area was maintained along the simulation where the HA-SP complex furnished several strong compact hydrogen bonding with the PC polar head. Such type of packing permitted the SPH complex to acquire an inverted cone structure with maintained micellar configuration. Finally, it was concluded that the composition of solvents could modify the packing parameters (Figure 2(d)) as well as the stability of the SPH ternary complex being identified for the adopted chloroform–ethanol solvating formulation.

### 3.2. Experimental design

d-Optimal design resulted in 15 formulae. The composition of the formulae is listed in Table 3.

**Table 3.** Experimental runs, independent variables and measured responses of SP- HAECs.

Formula^a^	Composition			*Y*_1_: EE%	*Y*_2_: PS (nm)	*Y*_3:_ PDI
	*X*_1_:Ceramide amount (mg)	*X*_2_: HA (mg)	*X*_3_: EA type			
HAEC 1	15	5	Kolliphor RH40	84.65 ± 1.36	329.61 ± 1.55	0.458 ± 0.009
HAEC 2	10	10	Kolliphor RH40	75.09 ± 2.08	300.00 ± 7.00	0.601 ± 0.031
HAEC 3	10	5	Kolliphor RH40	68.82 ± 1.19	319.00 ± 74.03	0.643 ± 0.094
HAEC 4	5	5	Kolliphor RH40	71.59 ± 5.13	350.00 ± 20.00	0.462 ± 0.057
HAEC 5	15	15	Kolliphor RH40	93.54 ± 2.06	297.30 ± 8.62	0.639 ± 0.049
HAEC 6	15	15	Kolliphor RH40	96.76 ± 1.30	292.60 ± 10.54	0.646 ± 0.042
HAEC 7	5	15	Kolliphor RH40	83.40 ± 1.20	297.30 ± 5.05	0.474 ± 0.025
HAEC 8	5	15	Kolliphor RH40	81.18 ± 1.96	292.60 ± 6.08	0.471 ± 0.020
HAEC 9	5	5	Kolliphor EL	44.78 ± 1.02	258.80 ± 2.86	0.489 ± 0.007
HAEC 10	5	5	Kolliphor EL	43.55 ± 1.45	261.8 ± 1.90	0.481 ± 0.005
HAEC 11	10	7.5	Kolliphor EL	68.23 ± 14.75	320.00 ± 4.00	0.455 ± 0.019
HAEC 12	10	15	Kolliphor EL	75.89 ± 16.38	340.10 ± 1.62	0.762 ± 0.023
HAEC 13	5	15	Kolliphor EL	68.76 ± 1.07	292.40 ± 14.50	0.587 ± 0.110
HAEC 14	15	15	Kolliphor EL	70.51 ± 0.89	459.33 ± 18.87	0.652 ± 0.077
HAEC 15	15	5	Kolliphor EL	70.29 ± 7.88	400.50 ± 20.50	0.612 ± 0.043

Presented values are the mean ± SD (*n* = 3).

^a^All formulae contained 10 mg SP and 100 mg phosphatidylcholine, in a volume of 10 mL.

Abbreviation: HA, hyaluronic acid; EA, edge activator; EE%, entrapment efficiency percent; PS, particle size; PDI, polydispersity index; SP, spironolactone; HAECs, hyaluronic acid enriched cerosomes.

### 3.3. Preparation of SP-HAECs using ethanol injection method employing D-optimal design

The preparation of SP-HAECs using ethanol injection method was successful. The resultant dispersions appeared white in color. The measured responses of HAEC 1–HAEC 15 are shown in Table 3. Owing to its insignificance, PDI was excluded from the optimization process.

### 3.4. Evaluation of PDI results

It’s well known that PDI values close to 0 indicate size homogeneity, while values close to 1 signify sample heterogeneity (Zeisig et al., 1996). The recorded values (Table 3) ranged from 0.455 ± 0.019 to 0.762 ± 0.023. These concluded the relative heterogeneity of the measured samples (Aburahma & Abdelbary, 2012).

### 3.5. Statistical analysis of the D-optimal design

Adequate precision assures the ability of model to navigate the design space when the measured signal to noise ratio is greater than 4, which was observed in all responses (De Lima et al., 2011). On the other hand, the predicted *R*^2^ is a measure of design’s ability to predict values of different responses (Chauhan & Gupta, 2004). The predicted and adjusted R^2^ values were in acceptable agreement (Table 4), ensuring there were no problems with the data or the model (Kaushik et al., 2006; Annadurai et al., 2008).

**Table 4.** Output data of the D-optimal design analysis of SP-HAECs.

Response	*Y*_1_: EE% (%)	*Y*_2_: PS (nm)
Minimum	43.6	258.8
Maximum	96.8	459.3
Ratio	2.22	1.77
Model	Linear	Quadratic
*R*-squared	0.9238	0.9970
Adjusted *R*-squared	0.9030	0.9930
Predicted *R*-squared	0.8545	0.9705
Adequate precision	19.643	57.726
Significant terms (coded factors)^a^	A, B, and C	A, C, AB, AC, BC, A^2^
Regression equation (in terms of actual factors)	EA type Kolliphor RH40 EE% = +50.92217 +1.46752 × Ceramide amount(mg) +1.50436 × HA amount(mg)	EA type Kolliphor RH40 PS(nm) = +458.60178 −26.42834 × Ceramide amount(mg) +0.48737 × HA amount(mg) +0.24540 × Ceramide amount(mg) × Hyaluronic acid amount(mg) +1.14427 × Ceramide amount(mg)^2^ −0.38439 × HA amount(mg)^2^
	EA type Kolliphor EL EE% = +34.86665 +1.46752 × Ceramide amount(mg) +1.50436 × HA amount(mg)	EA type Kolliphor EL PS(nm) = +238.93537 −10.09234 × Ceramide amount(mg) +9.48687 × HA amount(mg) +0.24540 × Ceramide amount(mg) × Hyaluronic acid amount(mg) +1.14427 × Ceramide amount(mg)^2^ −0.38439 × HA amount(mg)^2^

^a^A, Ceramide amount; B, HA amount; C, EA type.

Abbreviation: HA, hyaluronic acid; EA, edge activator; EE%, entrapment efficiency percent; PS, particle size; HAECs, hyaluronic acid enriched cerosomes.

#### 3.5.1. Effect of different variables on EE%

**Figure 3.**
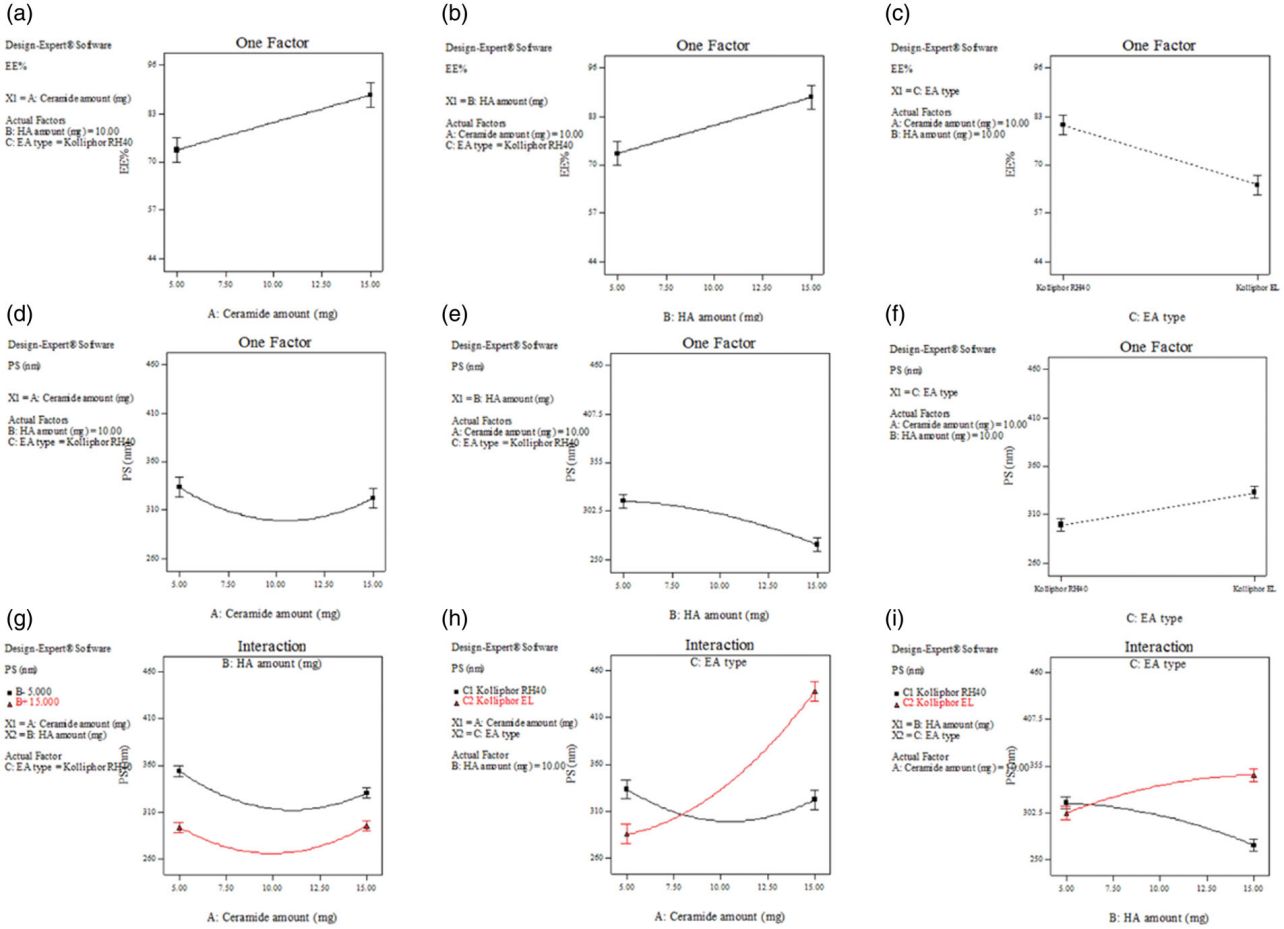
Effect of formulation variables on EE% of SP-HAECs (a–c), PS (d–f), while (g–i) show the effect of significant interactions on PS. Abbreviations: EE%, Entrapment efficiency percentage; PS, Particle size; SP, spironolactone; HAECs, Hyaluronic acid enriched cerosomes.

The values of EE% ranged from 43.55 ± 1.45 to 96.76 ± 1.30% (Table 3). The output data (Table 4), and Figure 3(a–c) show the effect of the studied variables on the EE% of the HAECs. All three factors, ceramide amount (*X*_1_), HA amount (*X*_2_) and EA type (*X*_3_) significantly affected the EE% of the prepared formulae (*p* = 0.0002 for ceramide amount and *p* < 0.0001 for HA amount and type of EA). The direct relation between ceramide amount and EE% could be attributed to the increased viscosity of formulation accompanying the increase in the amount of ceramide included and the consequent hinderance of diffusion of SP, ensuring that SP stay entrapped within the vesicles (Abdelgawad et al., 2017; Yousry et al., 2019). A previous study demonstrated the positive correlation between ceramide amount in formulation and fenticonazole nitrate EE% within PEGylated cerosomes (Albash et al., 2021). In addition to its contribution the overall viscosity of the medium, HA is known to augment the stiffness of the vesicles, thus counteracting the drug leakage that may occur due to the presence of EAs in HAECs. These combined effects, together with the results of the *in-silico* study, can explain the positive impact of HA amount on EE% (Manca et al., 2015; Sadeghi Ghadi et al., 2019). The results obtained in our study agree with Sadeghi-Ghadi et al. (2021) who reported that the presence of HA in niosomal formulations yielded high EE% of quercetin. With respect to the EA type, it was obvious that HAECs prepared using kolliphor RH 40 gave higher EE% than those prepared using kolliphor EL. The unsaturation sites in the alkyl chains of kolliphor EL may have increased the vesicle membrane permeability, thus lowering EE% (Nemr et al., 2021; Mokhtar et al., 2008; Al-Mahallawi et al., 2015). These results agree with Abdelbary et al. who found that bilosomes decorated with kolliphor RH 40 entrapped more terconazole than those decorated with kolliphor EL (Abdelbary et al., 2016).

#### 3.5.2. Effect of different variables on PS

The PS values ranged from 258.80 ± 2.86 to 459.33 ± 18.87 nm (Table 3). The output data (Table 4) and Figure 3(d–f) show the effect of the studied variables on the PS of the HAECs. Both ceramide amount (*X*_1_) and EA type (*X*_3_) significantly affected the PS (*p* < 0.0001 for both factors). On the other hand, HA amount (*X*_2_) had no significant effect (*p* = 0.3541). The direct relation between ceramide amount and PS can be explained in the light of two reasons. The first of which is that ceramides tend to aggregate in formulation. The second one is the ceramide induced changes in the vesicles’ membranes. Ceramides have limited ability to cross between the vesicle membrane leaflets, leading to its accumulation within them. The overall outcome is an increase in PS (Castro et al., 2014; Albash et al., 2021). HAECs decorated with kolliphor RH 40 had smaller PS than those decorated with kolliphor EL. Both EAs are composed of hydrophilic and hydrophobic counterparts, with the hydrophilic counterpart being responsible for prevention of vesicular aggregation (Chong et al., 2011). The hydrophilic counterparts of both EAs are composed of polyethylene oxide (PEO) units. Kolliphor RH 40 has 40 PEO units, while kolliphor EL contains only 35 PEO units (Madheswaran et al., 2014). Therefore, it is logical that the former one has more stabilizing ability and thus its employment has led to smaller PS. The observed results agree with Abdelbary et al., (2016).

Based upon the abovementioned, different interactions have also been found significant (Figure 3(g–i)). The interaction between ceramide amount and HA amount (AB) had *p* value equal to 0.0044, where the smallest PS were obtained by using intermediate levels of both factors. Also, increasing HA amount for the same ceramide amount yielded smaller PS (Figure 3(g)). In addition, the interaction between ceramide amount and EA type (AC) had *p* < 0.001. Increasing ceramide amount while using kolliphor RH 40 did not much affect PS, with the smallest sizes obtained using an intermediate ceramide level. On the contrary, increasing ceramide amount while using kolliphor EL dramatically increased PS with the smallest sizes obtained using the lowest possible ceramide level (Figure 3(h)). Moreover, the interaction between HA amount and EA type had *p* < 0.0001. Increasing HA amount while using kolliphor RH 40 decreased PS. The opposite was observed upon using kolliphor EL (Figure 3(i)).

### 3.6. Optimization of SP-HAECs based on the desirability criterion

SP-HAECs were to be optimized according to the constraints listed in Table 2 with the exclusion of the non-significant response (PDI). The optimum values of the variables were obtained by numerical optimization based on the criterion of desirability using the Design-Expert-7^®^ software (Basalious et al., 2010). A suggested OHAEC containing 10.5 mg ceramide and 15 mg HA, utilizing Kolliphor RH40 as EA had the highest desirability of 0.956 (Supplementary Figures S1 and S2). The observed EE% and PS results were 89.3 ± 0.3% and 261.8 ± 7.0 nm, respectively. The predicted values were 88.9% and 266.7 nm, respectively. The bias percent was 0.45 and 1.87%, for EE % and PS, respectively. The high similarity between the observed and predicted responses of OHAEC could conclude the validity of the design to predict the responses.

### 3.7. Further *in vitro* characterization of OHAECs formula

#### 3.7.1. Effect of short-term storage

The values of EE%, PS, ZP, and PDI for fresh and stored OHAEC are listed in Table 5. Supplementary Figures S3 and S4 demonstrate the PS and ZP of OHAEC, respectively after storage. No statistical difference was found in EE%, PS, ZP, and PDI (*p* > 0.05 for all values). Moreover, there was no change in the physical appearance of OHAEC.

**Table 5.** Effect of storage on different measurements of OHAEC.

Parameter^a^	Fresh OHAEC	Stored OHAEC (3 months)
EE%	89.3 ± 0.3	88.5 ± 1.6
PS (nm)	261.8 ± 7.0	267.8 ± 10.1
ZP (mV)	−9.0 ± 1.1	−7.3 ± 2.4
PDI	0.482 ± 0.07	0.465 ± 0.06

^a^Mean ± SD (*n* = 3).

Abbreviation: EE%, entrapment efficiency percent; PS, particle size; PDI, polydispersity index; ZP, zeta potential; OHAECs, optimal hyaluronic acid enriched cerosomes.

#### 3.7.2. Morphology of SP-OHAEC

**Figure 4.**
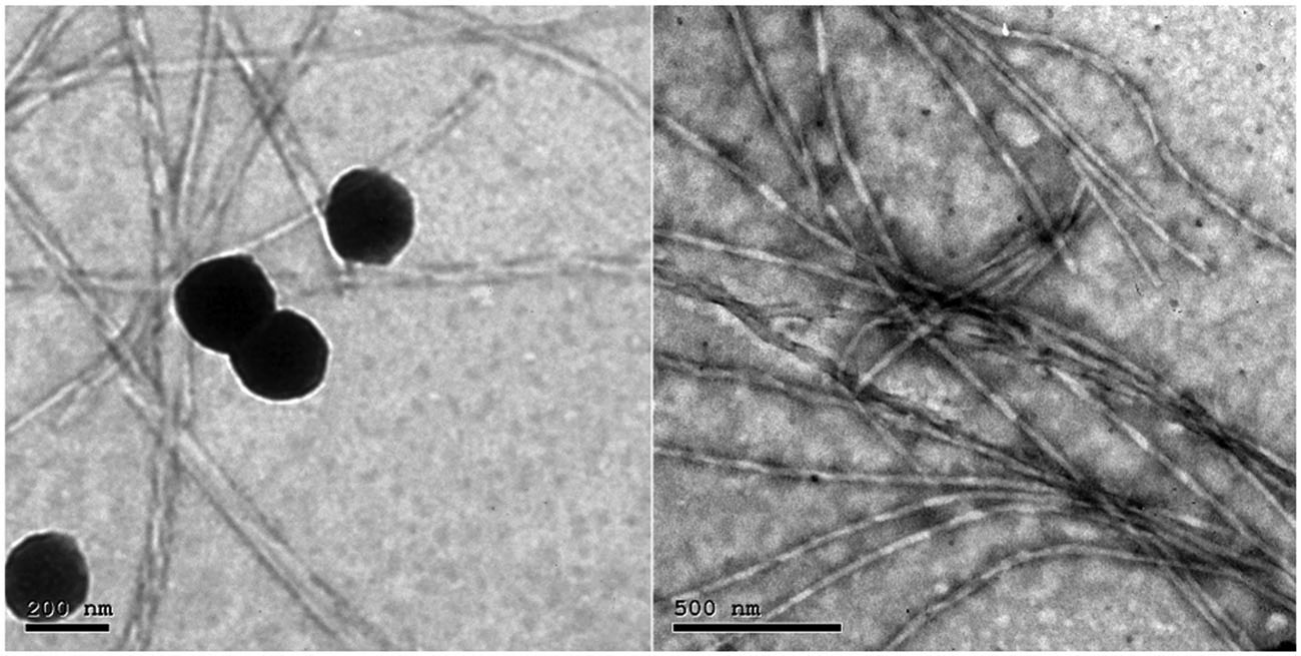
Transmission electron micrographs of SP OHAEC showing mixed vesicular and tubular appearances. Abbreviation: OHAEC, optimal hyaluronic acid enriched cerosomes.

It is obvious in Figure 4 that ceramide has led to a change in morphology of particles from the ordinary vesicular structure into an elongated tubulated form. This could be attributed to the fact that enriching PC with ceramide caused elongation of their vesicles attributed to the partitioning of ceramide into the PC bilayer accompanied by rigidification of the interface. The high packing parameter of ceramide compared to that of the PC caused flattening of the PC bilayer curvature upon vesicles preparation. The occasional presence of spherical vesicles along with the tubules may be due the non-uniform distribution of ceramide in the bilayer, resulting in ceramide rich domains with a flat morphology and ceramide poor domains with spherical morphology. These findings were in agreement with Xu et al. (2009) and Abdelgawad et al. (2017) upon the preparation of ceramide VI-based cerosomes, and Albash et al. (2021), upon the preparation of ceramide IIIB-based cerosomes.

### 3.8. *Ex vivo* permeation and deposition studies

**Figure 5.**
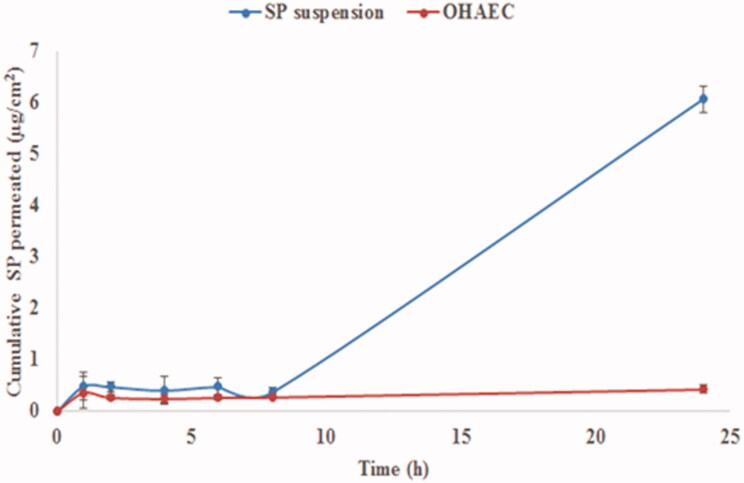
*Ex vivo*profile of SP from OHAEC, compared to its aqueous suspension. Abbreviation: SP: spironolactone, OHAEC: optimal hyaluronic acid enriched cerosomes.

It is obvious that encapsulation of SP within OHAEC has led to its retention, limiting its permeation compared to its suspension (Figure 5). This was reflected by its significantly lower *Q*_24_ (cumulative amount permeated per unit area after 24 h) and *J*_max_ values and significantly higher Dep_24_ value leading to LAEI value 18.5 times higher than suspension (*p* < 0.05) (Table 6). Formulation components, HA and ceramide III are known to enhance the localization of drugs, while limiting their permeation to blood. A recently marketed formula of diclofenac in HA-gel base has shown acceptable action with minimal amounts detected in blood (Brown and Jones, 2005). On the other hand, ceramides are known to interact with keratin in corneocytes, allowing for better fusion between the PC in the formula and skin components, leading to drug localization (Abdelgawad et al., 2017; Albash et al., 2021).

**Table 6.** *Ex vivo* permeation and deposition parameters of OHAEC, compared to SP suspension.

Parameter^a^	OHAEC	SP-suspension
Q_24_ (µg/cm^2^)	0.43 ± 0.08	6.07 ± 0.26
Dep_24_ (µg/cm^2^)	55.45 ± 0.35	42.74 ± 5.82
*J*_max_ (µg/h cm^2^)	0.018 ± 0.003	0.253 ± 0.011
LAEI	129.78 ± 11.22	7.02 ± 0.66
LAEI ratio	18.48	–

^a^Mean ± SD (*n* = 3).

Abbreviation: Q_24_, cumulative amount permeated per unit area after 24 h; Dep_24_, cumulative amount deposited per unit area after 24 h; *J*_max_, maximum average flux after 24 h; LAEI, Local accumulation efficiency index; SP, spironolactone; OHAECs, optimal hyaluronic acid enriched cerosomes.

### 3.9. *In vivo* studies

#### 3.9.1. Histopathological studies

**Figure 6.**
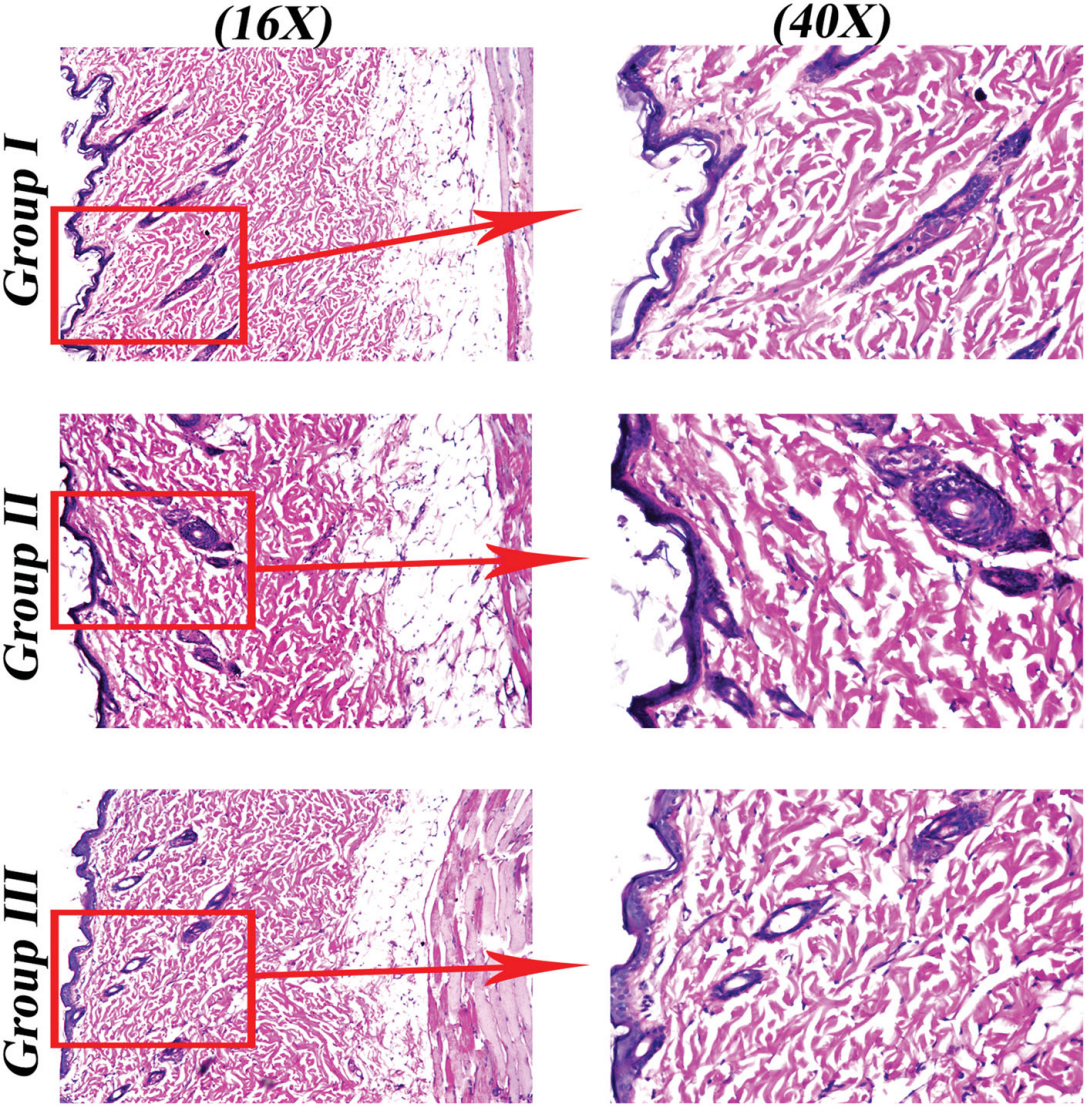
Light microscope photomicrographs showing histopathological sections (hematoxylin and eosin stained) of rat skin normal control (group I), rat skin treated with SP suspension (group II) and rat skin treated with OHAEC (group III) with magnification power of 16X to illustrate all skin layers (Left side) and magnification power of 40X to identify the epidermis and dermis (Right side). Abbreviation: SP: spironolactone, OHAEC: optimal hyaluronic acid enriched cerosomes.

Figure 6 revealed that rats treated with SP suspension (group II) and OHAEC (group III) did not show any histopathological alternations in rats’ skin compared to untreated skin sections (group I). These findings support the tolerability of OHAEC for topical application.

#### 3.9.2. Dermatokinetic study

**Figure 7.**
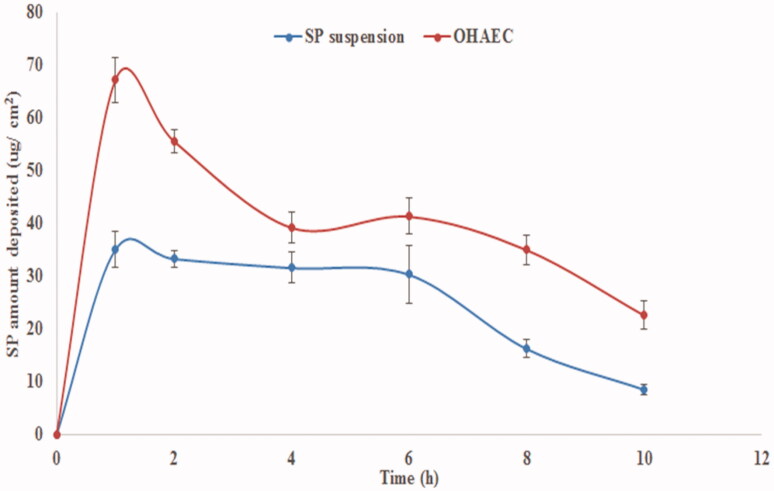
*In vivo* skin deposition profile of SP from OHAEC, compared to its aqueous suspension. Abbreviation: SP: spironolactone, OHAEC: optimal hyaluronic acid enriched cerosomes.

The deposition profile of SP from OHAEC, compared to SP suspension is illustrated in Figure 7. SP was deposited from OHAEC in higher amounts compared to that from the suspension. The AUC_0–10_, obtained from the deposition profiles was 402.9 µg h/cm^2^ for OHAEC, which was significantly higher than that of suspension (248.3 µg h/cm^2^), meaning a 1.62 folds increase in SP-deposition. These findings agree with findings of the *ex vivo* study and signify the role of formulation components in localizing SP within the skin.

## 4. Conclusions

SP-loaded HAECs were prepared by ethanol injection, according to D-optimal design, following an *in silico* study that exhibited the importance of hyaluronic acid in increasing the extent of binding between SP and phospholipid. Optimal HAECs (OHAEC) contained 10.5 mg ceramide III, 15 mg HA and Kolliphor RH40 as an edge activator. OHAEC was stable for up to 3 months. Transmission electron microscopy showed a mixed tubular and vesicular appearance of OHAEC. Both *ex vivo* and *in vivo* dermatokinetic studies concluded better deposition of SP from OHAEC, compared to its suspension. Histopathology demonstrated the safety of OHAEC. Hence, it could be concluded that OHAEC could be effective formula that could treat hirsutism.

## Supplementary Material

Supplemental Material
