# Supplementary material for: Spironolactone hyaluronic acid enriched cerosomes (HAECs) for topical management of hirsutism: in silico studies, statistical optimization, ex vivo, and in vivo studies
Source: Drug Deliv. 2021 Nov 2;28(1):2289–300. doi: 10.1080/10717544.2021.1989089 (PMC8567875; doi:10.1080/10717544.2021.1989089)
Supplement: Supplemental Material [file IDRD_A_1989089_SM8280.pdf]

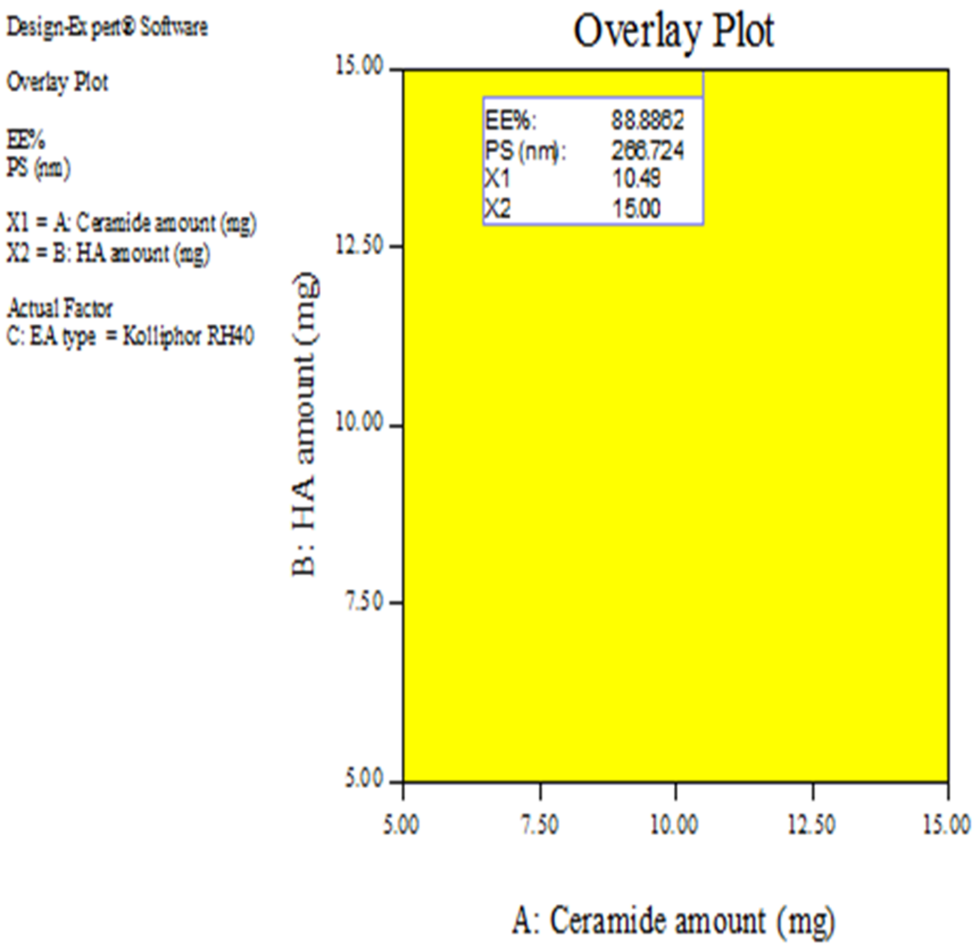

Supplementary figure 1: Overlay plot for the optimization of SP HAECs.

SP: Spironolactone, HAECs: Hyaluronic acid enriched cerosomes.

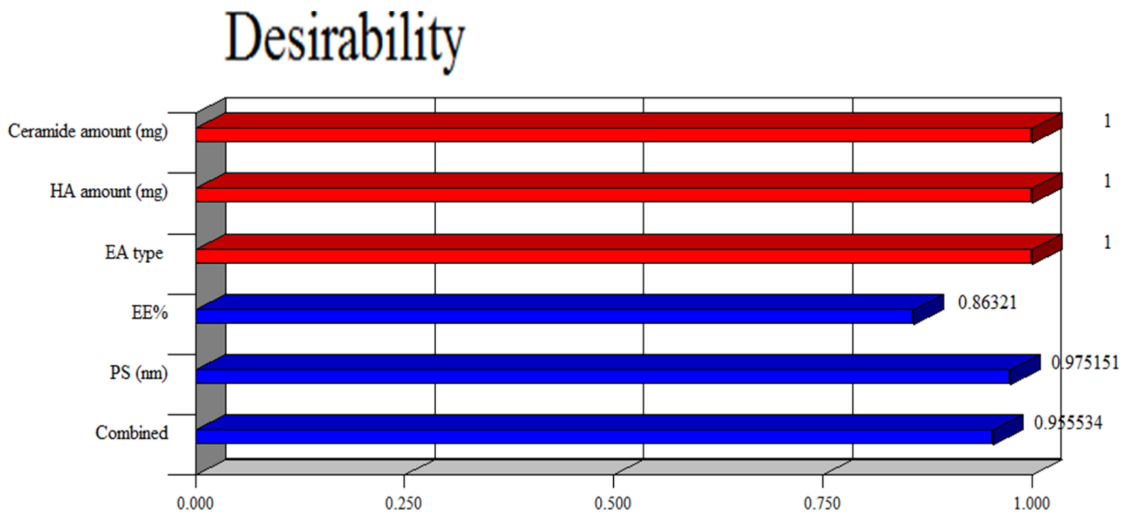

Supplementary figure 2: Desirability bar plot showing desirability values of design factors, individual responses and combined responses for SP OHAEC.

SP: spironolactone, OHAEC: optimal hyaluronic acid enriched cerosomes.

Size Distribution Report by Intensity

v2.2

Malvern

Sample Details

Sample Name:	F2 3
SOP Name:	mansettings.nano
General Notes:	

File Name:	Dr.Rania	Dispersant Name:	Water
Record Number:	176	Dispersant RI:	1.330
Material RI:	1.59	Viscosity (cP):	0.8872
Material Absorbtion:	0.010	Measurement Date and Time:	Thursday, June 10, 2021 1:44...

System

Temperature (°C):	25.0	Duration Used (s):	80
Count Rate (kcps):	166.1	Measurement Position (mm):	4.65
Cell Description:	Disposable sizing cuvette	Attenuator:	8

Results

			Size (d.nm):	% Intensity:	St Dev (d.n...
Z-Average (d.nm):	267.8	Peak 1:	290.9	88.1	105.2
PdI:	0.465	Peak 2:	77.38	11.9	16.75
Intercept:	0.995	Peak 3:	0.000	0.0	0.000
Result quality :	Good				

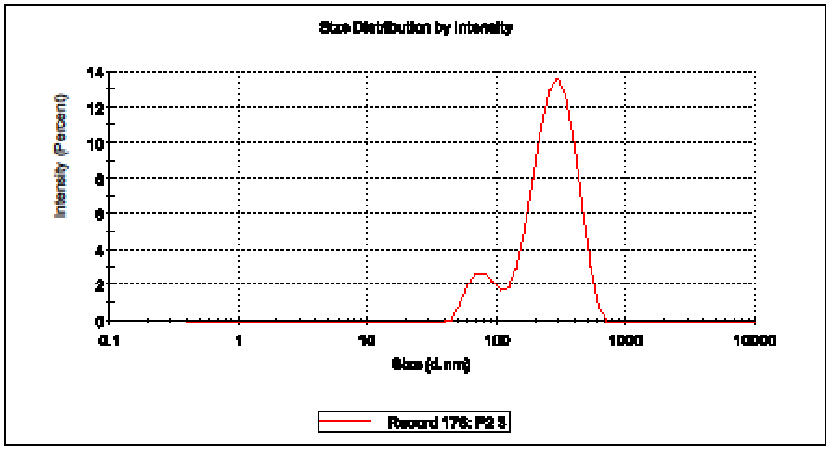

Supplementary figure 3: Zeta sizer size report for OHAEC size stability.

OHAEC: optimal hyaluronic acid enriched cerosomes

Zeta Potential Report

v2.3

Malvern Instruments Ltd - © Copyright 2008

Malvern

Sample Details

Sample Name: F2 3

SOP Name: mansettings.nano

General Notes:

**File Name:**	Dr.Rania	**Dispersant Name:**	Water
**Record Number:**	122	**Dispersant RI:**	1.330
**Date and Time:**	Thursday, June 10, 2021 1:59:40 AM	**Viscosity (cP):**	0.8872
		**Dispersant Dielectric Constant:**	78.5

System

**Temperature (°C):**	24.9	**Zeta Runs:**	12
**Count Rate (kcps):**	158.8	**Measurement Position (mm):**	2.00
**Cell Description:**	Clear disposable zeta cell	**Attenuator:**	10

Results

			**Mean (mV)**	**Area (%)**	**St Dev (mV)**
**Zeta Potential (mV):**	-7.30	**Peak 1:**	-7.30	100.0	3.45
**Zeta Deviation (mV):**	3.45	**Peak 2:**	0.00	0.0	0.00
**Conductivity (mS/cm):**	0.444	**Peak 3:**	0.00	0.0	0.00
**Result quality :**	Good				

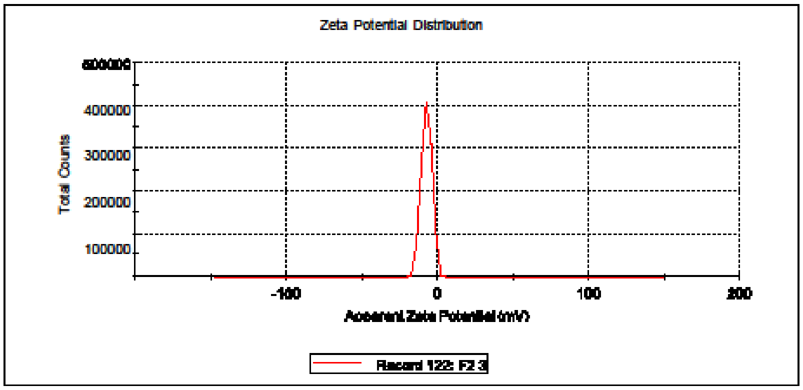

Supplementary figure 4: Zeta sizer zeta potential report for OHAEC zeta potential stability.

OHAEC: optimal hyaluronic acid enriched cerosomes
